# Spatio‐temporal variation in reproductive characteristics of invasive fish *Pseudorasbora parva* (Temmick & Schlegel, 1846) in the lakes region of Türkiye

**DOI:** 10.1111/jfb.15932

**Published:** 2024-10-09

**Authors:** Fahrettin Küçük, Ergi Bahrioğlu, Salim Serkan Güçlü, Mevlüt Nazıroğlu, İskender Gülle, Sera Övgü Kabadayı Yıldırım

**Affiliations:** ^1^ Isparta University of Applied Sciences, Eğirdir Fisheries Faculty Isparta Turkey; ^2^ Burdur Mehmet Akif Ersoy University, Faculty of Science and Literature, Department of Biology Burdur Turkey

**Keywords:** fecundity, gonadal histology, invasive species, sexual cycle, stone moroko

## Abstract

Invasive species present a serious peril to aquatic ecosystems worldwide, thus it is essential to have a comprehensive understanding of the reproductive dynamics, spreading characteristics, and biological properties of these species in order to effectively manage their population structure and mitigate both the ecological damage and economic loss they can cause. For this reason, we delved into the reproductive dynamics of *Pseudorasbora parva*, an invasive species of inland water fish found in Türkiye. We focused on three populations inhabiting Beyşehir (Konya, Isparta) and Eğirdir Lakes (Isparta) and Onaç Reservoir (Burdur). Sampling was carried out on a monthly basis from March 2021 to June 2022, utilizing a variety of tools such as multimesh nets, seine nets, and electrofishing. The developmental stages of gonads and reproductive cells were determined through morphological and microscopic examinations. A total of 1186 *P. parva* individuals were captured from all three lakes. Our results showed that the Beyşehir Lake population spawned from March to July, while the Eğirdir Lake population did so between May and October, and the Onaç Reservoir population laid their eggs from June to August. Female individuals in Beyşehir Lake, Eğirdir Lake, and Onaç Reservoir had initial breeding lengths of 3.49, 4.89, and 5.35 cm, respectively. In comparison, male individuals measured 5.56, 4.80, and 5.40 cm. Interestingly, the Beyşehir Lake population exhibited the highest egg fecundity, with each individual at 2 years producing a remarkable 2949 eggs. The present findings provide valuable information for us to further understand the reproductive biology and dynamics of *P. parva*, which could be useful in managing and preventing the spread of this invasive species in inland aquatic ecosystems.

## 1 INTRODUCTION

The majority of teleost fish species, including the invasive *Pseudorasbora parva*, exhibit iteroparity, suggesting they have the ability to undergo many reproductive cycles during their lifespan. Some bioecological characteristics, such as short lifespans, extended reproductive periods, high reproductive capacity, and parental care, are important factors that allow teleost fish species to successfully invade new habitats (Ekmekçi & Kırankaya, 2004, 2006). *P. parva* has been reported to have a high rate of reproductive success due to its small size, diverse range of food sources, high ecological tolerance, sporadic egg laying, high fecundity of females, and protection of eggs by males (Britton et al., 2007; Kirczuk et al., 2021). Furthermore, research has shown that *P. parva* has a broad physiological tolerance allowing it to survive pesticide concentrations that would be lethal to other organisms (Švolíková et al., 2016). *P. parva* is a small cyprinid species indigenous to Japan, China, Korea, and the Amur River basin. Since its accidental introduction to Europe in 1961, it has rapidly expanded its range to include North Africa (Algeria, Morocco), the Middle East (Türkiye, Iran), Central Asia (Kazakhstan, Uzbekistan), and even Fiji in Oceania (Gozlan et al., 2005; Kirczuk et al., 2021; Welcomme, 1988). This data provides important clues that *P. parva* might be transported via anthropogenic activities to distant areas outside of its indigenous continent and then invade those areas.

*P. parva* is found in a wide variety of habitats, mostly in well‐vegetated small waterways, ponds, and lakes. The accidental introduction of this species to new bodies of water frequently occurs during the process of stocking other fish species (Wildekamp et al., 1997). Studies on *P. parva* populations in the Danube River Basin and Lake Lichensk have shown that females become sexually mature when they reach a typical length of 20–25 mm. This is attributed to their ability to adapt to different environments and rapidly spread to new habitats, suggesting they exhibit high phenotypic plasticity (Kirczuk et al., 2021; Švolíková et al., 2016). *P. parva* has rapidly invaded Wardynka River (Poland), leading to an increase in both population size and density depending on its copious egg production, sporadic spawning pattern, and prolonged 4‐month breeding time in a newly colonized environment (Kirczuk et al., 2021). *P. parva* possesses a high reproductive potential and bioavailability, which enables it to proliferate and occupy new habitats. Due to its inherent hallmarks, *P. parva* poses a serious threat to other species present in aquatic ecosystems (Kirczuk et al., 2021).

A study on the Danube River Basin (Bulgaria) and Lake Lichensk (Poland) revealed that female individuals reached sexual maturity at 20–25 mm standard length (SL) in the Danube basin. These results provide valuable insights into the invasive and reproductive potential of *P. parva* populations (Švolíková et al., 2016). Furthermore, the overall absolute fecundity reached the maximum rate documented for this species, regardless of whether native or non‐native. Their findings also indicate that *P. parva* exhibits a remarkably high capacity for producing oocytes as well as significant phenotypic plasticity and bioavailability. This suggests that *P. parva* has the potential to spread and propagate in other invasion regions (Švolíková et al., 2016). Furthermore, *P. parva* has somewhat smaller testicles compared to other cyprinids. However, its distinct characteristics, such as nest protection behavior and extended breeding periods, have been reported to improve reproductive success and increase the survival of newly hatched larvae. Moreover, its remarkable capacity to reproduce and thrive in diverse environmental circumstances and habitats has been documented as a key factor in its successful spread in new locations (Kirczuk et al., 2021).

After its initial record in the Meriç River in Türkiye in 1982 (Erk'akan, 1984), *P. parva* was found in the Aksu Stream in the Antalya basin in 1993. Wildekamp et al. (1997) then documented its interior distribution in Anatolia. This invasive species is currently prevalent in a large area that spans from Thrace to the Ceyhan basin. Recent data shows that its range extends as far east as the Aras and Kura basins (Küçük & Güçlü, 2017). This study aimed to evaluate the reproductive dynamics and biological properties of *P. parva* populations inhabiting Beyşehir Lake, Eğirdir Lake, and Onaç Reservoir, all of which exhibit distinct ecological aspects. The present study also investigated the distribution and reproductive potential of this invasive species.

## 2 MATERIALS AND METHODS

### 2.1 Study area and sampling

Our sampling areas consisted of Eğirdir Lake at 38°08′44.56″N, 30°46′38.75″E, Beyşehir Lake at 37°43′10.11″N, 31°40′04.36″E, and Onaç Reservoir at 37°29′52.74″N, 30°34′04.89″E. During monthly sampling program set up from March 2021 to June 2022, we used multimesh gill nets (10, 15, 20, 40, 55, 70, 80, and 100 mm) specifically designed for stagnant water habitats. We conducted additional samplings with the help of a 4‐mm mesh net (10 m in width and 1.5 m in depth) and an electroshocker through the coastal regions of those lakes. A seine net with a mesh size of 4 mm (1.5 m in depth and 10 m in length) was also utilized in the littoral zones of those lakes in addition to this fishing equipment.

A total of 1186 *P. parva* individuals were collected, comprising 335 from Eğirdir Lake, 544 from Beyşehir Lake, and 307 from Onaç Reservoir. The fish samples were measured for their total lengths (TLs) using a digital caliper with a precision of 0.05 mm. Their live weights were determined using a digital scale with a precision of 0.01 g in a laboratory setting. We also estimated the age of the fish by examining scales obtained from the area between the left lateral region and the dorsal fin origin of the fish.

### 2.2 Gonadosomatic index and fecundity

The gonadosomatic indexes were calculated for each month using the formula GSI = GW/W × 100, where GW denotes the weight of the gonads and W denotes the total body weight. The fecundity of mature female individuals was assessed by dissecting both the right and left ovaries and measuring their weight using a digital scale (Gibson & Ezzi, 1980). The gravimetric method was employed to determine the number of mature oocytes in three distinct areas of the ovaries. The average diameters of the eggs were measured using a micrometer, as described by Nikolsky in 1980. The fecundity of a total of 111 mature female individuals comprising 48 from Beyşehir Lake, 46 from Eğirdir Lake, and 17 from Onaç Reservoir at the spawning stage was determined using the gravimetric method. We utilized the sigmoid logistic curve method to determine the maturity level of fully mature female individuals. We also applied the logistic methodology to estimate the initial reproductive length (*L*_50_). All calculations were conducted using the SizeMat R package (Torrejon‐Magallanes, 2019) in RStudio v1.2.5001 software (RStudio Team, 2015). In accordance with the methodology described by Sparre and Venema (1992), we calculated Fulton's condition factor (CF) via the formula (BW/TL^3^) × 100, where BW is body weight (g) and L is total length (cm).

### 2.3 Histological examinations

The female and male gonad samples were fixed in 10% formalin solution and embedded in paraffin. 5‐micrometer‐thick sections were cut from the paraffin‐embedded tissues and placed onto slides (SLEE Cut 4062). Microscopic sections were used to represent few regions of each gonad, with each section containing five to 15 pieces of the gonad. The hematoxylin–eosin staining method was employed for staining the tissue sections. The stained sections were examined under a light microscope (Olympus CX21) (Bancroft & Stevens, 1996).

We used the classification system specified by Domagała et al. (2013) to define the sexual cycle of female and male gonads. The development of oocytes can be categorized into six stages: stage I is characterized by the sexual differentiation of cells, stage II denotes oocytes in previtellogenesis, stages III and IV show oocytes undergoing vitellogenesis, stage V marks advanced vitellogenesis, which is crucial for maturation and reproduction, and stage VI represents the post‐spawning process of the ovary, where various types of oocytes can be observed. The diameter of eggs was carefully measured under a microscope (with an accuracy of 0.01 mm). Five oocytes with the longest and shortest diameters were harvested from each ovary.

Similarly, we used a modified six‐stage classification specified by Domagała et al. (2015) to describe the sexual cycle of male gonads. Stage I denotes the formation of type A spermatogonia in seminiferous tubules, whereas stage II marks the formation of type B spermatogonia in the same tubules. At stage III, primary spermatocytes start to appear in the seminiferous tubules and spermatogenesis continues. Cells spanning the whole range of spermatogenetic stages, from type A spermatogonia to spermatozoa, reside in the seminiferous tubules at stage IV. The tubule lumen occurs and there is an efferent duct filled with many spermatozoa. In stage V, the seminiferous tubules are filled with a large number of spermatozoa and the effluent duct is filled with milt, indicating about‐to‐complete spermatogenesis. Stage VI denotes the completion of nearly all stages of spermatogenesis, with the testicles entering a “spent condition.” The seminiferous tubules only contain a small amount of residual sperm, and there may be some left in the efferent duct.

## 3 RESULTS

### 3.1 GSI and the morphological stage of gonad development

**FIGURE 1:**
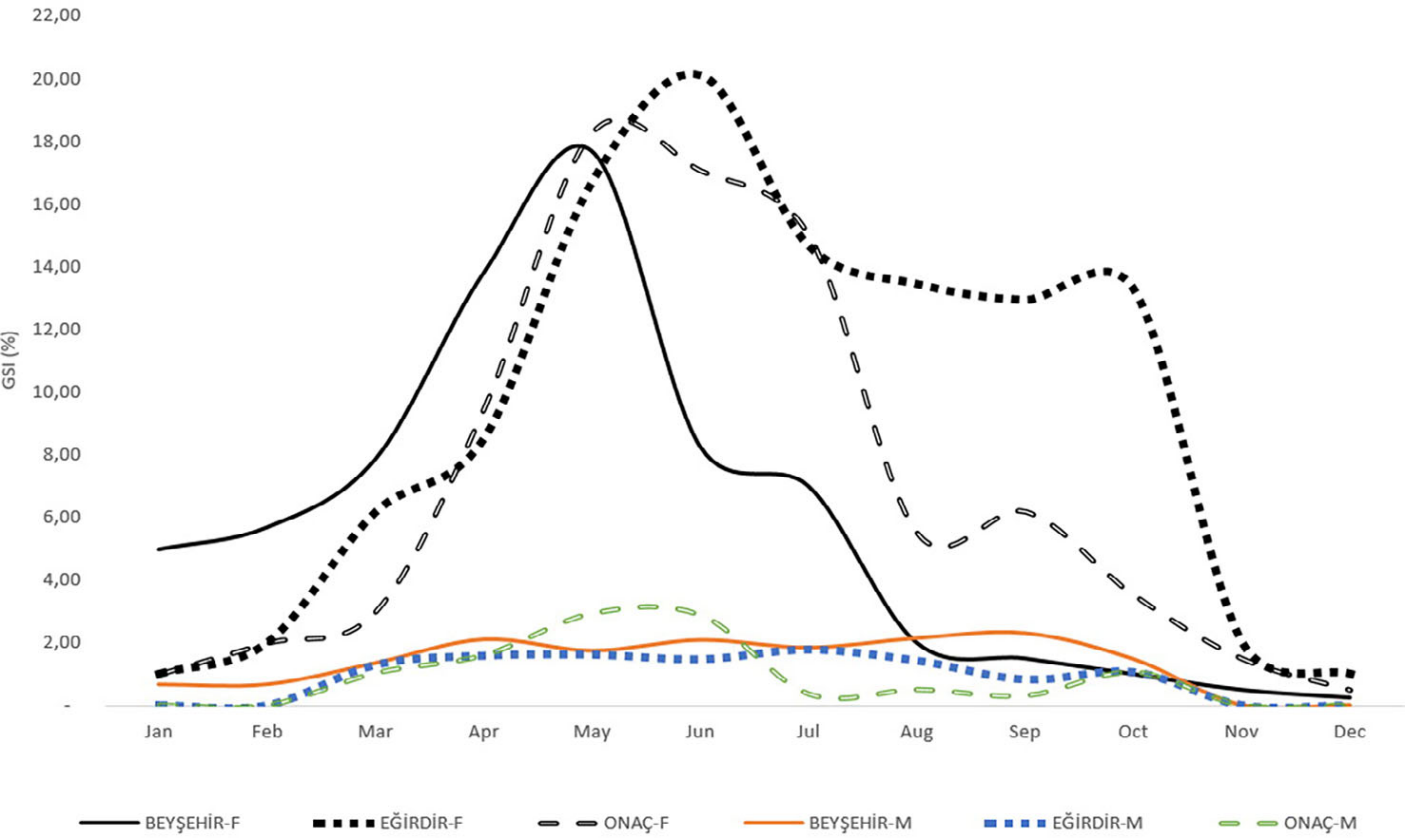
Monthly Gonadosomatic Index (GSI) values of the *Pseudorasbora parva* population in the Beyşehir and Eğirdir Lakes and Onaç Reservoir. F, female; M, male.

The reproductive period of the population in Beyşehir Lake was determined using data on 544 individuals of *P. parva*. The GSI values on the lake population exhibited a substantial increase from February to May, peaking at 17.68% at the end of May. Conversely, there was a sharp decline in the weight of the gonads in July and August, as depicted in Figure 1. It is evident from the changes in GSI values that the lake population spawns intermittently from March to July.

Data analysis on 335 *P. parva* individuals allowed us to determine the reproductive cycle of the population in Eğirdir Lake. The GSI data on the female population experienced a significant and rapid rise from March to June, reaching its maximum (20.09%) at the end of June. The GSI value decreased to 14.64%, with a partial decline in July and October compared to June (Figure 1). It can be inferred from the GSI values that the lake population exhibits sporadic egg‐laying behavior from May to October.

The analysis of 307 *P. parva* individuals from Onaç Reservoir showed that the GSI value of the population there increased rapidly to 18.29% between April and May. Despite a partial decrease in July and October, the gonad occupancy rates remained relatively high (17.07–15.01%) in June and July (Figure 1). The GSI data reveals that the Onaç Reservoir population lays eggs sporadically from May to October.

**FIGURE 2:**
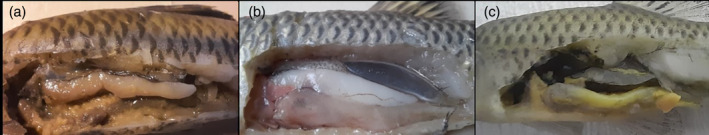
Morphological appearance of different testicular developmental stages in *Pseudorasbora parva* populations: (A) pre‐breeding period, (B) reproductive period, and (C) post‐reproductive period.

**FIGURE 3:**
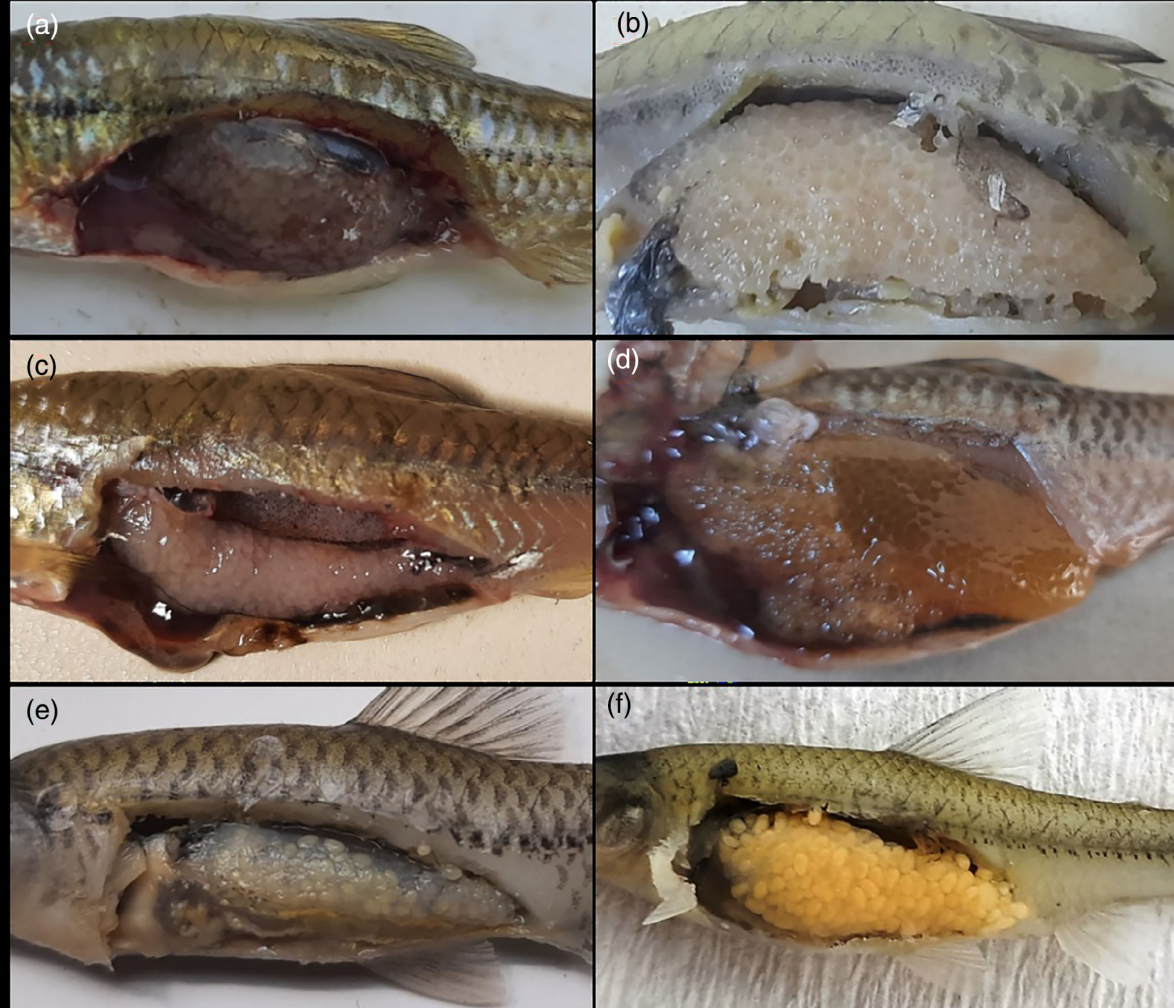
Morphological appearance of different ovarian developmental stages in *Pseudorasbora parva* populations: (A) mature female before the reproductive period (Eğirdir Lake), (B) mature female in the breeding period in Eğirdir Lake, (C) mature female before the reproduction period in Beyşehir Lake, (D) mature female during the breeding period in Beyşehir Lake, (E) mature female after the breeding season in Onaç Reservoir, and (F) mature female in the breeding season in Onaç Reservoir.

However, an external examination of the gonads showed that the population spawned intermittent from March to October (Figure 2). Macroscopic observations showed that ovarian development in all habitats has been at stage II since March (Figure 3). During May and June (stage IV), the ovaries exhibited their highest yield with observable mature eggs during samplings (Figure 3). It is important to note that the ovaries were empty after June, with oocytes at different developmental stages detected within the ovaries (Figure 3).

### 3.2 Histology of ovarian development

**FIGURE 4:**
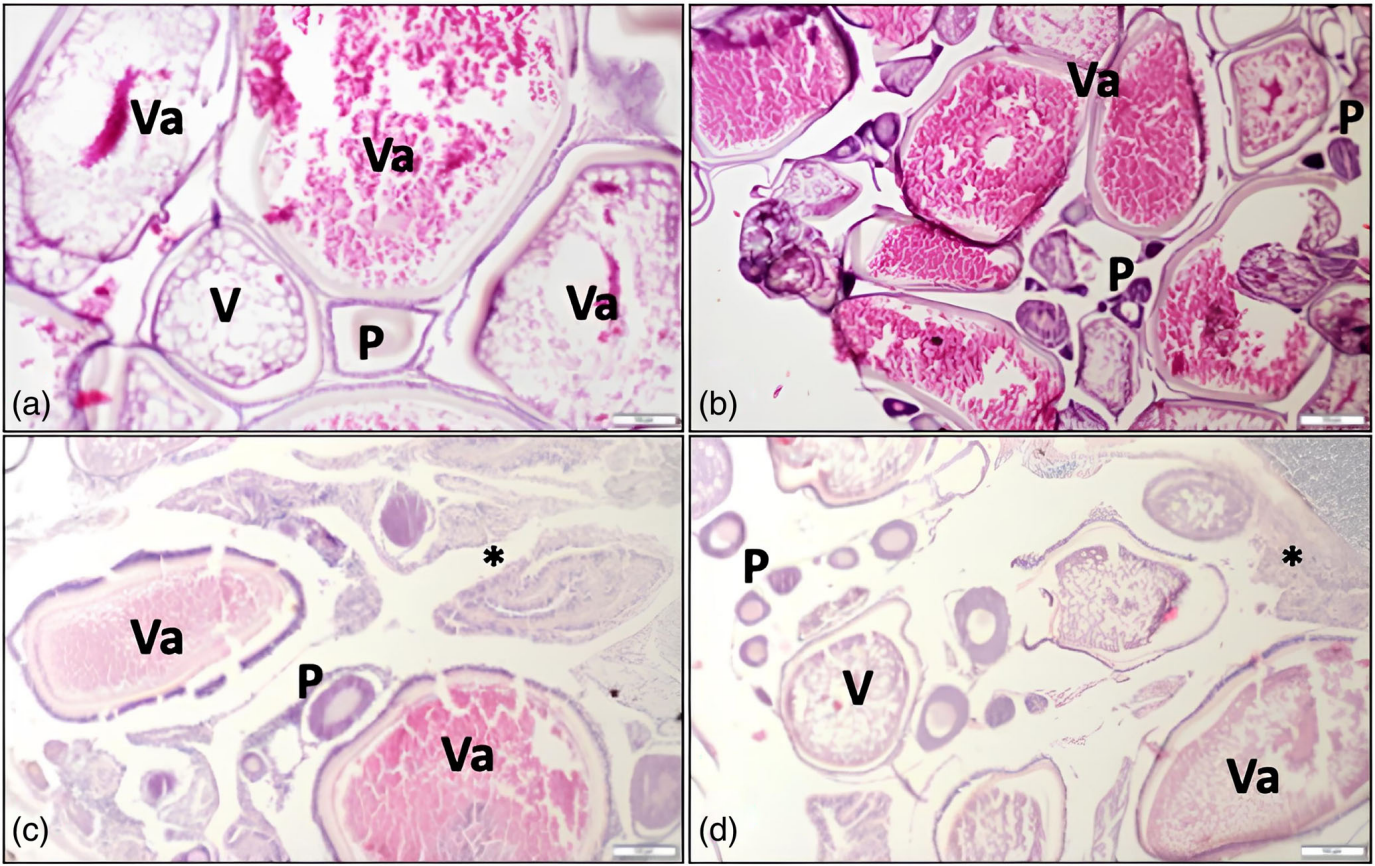
Gonad histology of *Pseudorasbora parva* female individuals. Reproductive gonads were observed throughout the entire season, along with spent gonads. (A) At the beginning of reproductive period, follicles of varying sizes were observed in stage III (IV) vitellogenesis. (B) In stage V, the majority of the population experienced advanced vitellogenesis, indicating the onset of reproduction. (C) Observation of post‐ovulatory follicles and oocytes indicates ongoing reproduction. (D) Stage VI: reproductive end. P, previtellogenesis; V, vitellogenesis; Va, advanced vitellogenesis; *post‐ovulation follicles.

In March, the population of female *P. parva* individuals in Beyşehir Lake was reported to be in stages II and III (IV). Numerous pre‐vitellogenic follicles were detected during the second stage of ovarian development. In stage III (IV), follicles of various sizes undergoing vitellogenesis were discovered, as shown in Figure 4. The population had the greatest proportion of stage V ovaries from mid‐April to mid‐May. During this period, there were individuals within the population prepared for intensive breeding (Figure 4). Stage VI reproductive and post‐reproductive gonads were visible from mid‐May to late June. This coincided with the vitellogenesis, vitellogenesis, and advanced vitellogenesis phases (Figure 4). Stage IV ovaries were observed in the Eğirdir Lake population between the second half of April and the first half of May (Figure 4a). Stage V ovaries were detected from mid‐May to late June (Figure 4). This period exhibited the most intense reproductive activities. Many females in the population had stage VI gonads from the first half of July to early October. During this period, some gonads also displayed various phases of vitellogenesis, as seen in Figure 4. The ovaries of female individuals in the Onaç Reservoir were in stage II (previtellogenesis) and stage III (IV) from early April to mid‐May (Figure 4). Reproductive gonads (V. stage) were found in most individuals in the second half of May (Figure 4). This stage was complete in the first half of July, and the ovaries transitioned to the post‐reproductive stage (VI. stage) (Figure 4). The histological examination revealed an increase in this stage in the Onaç Reservoir population from the second half of August to the end of October.

### 3.3 Histology of testicular development

**FIGURE 5:**
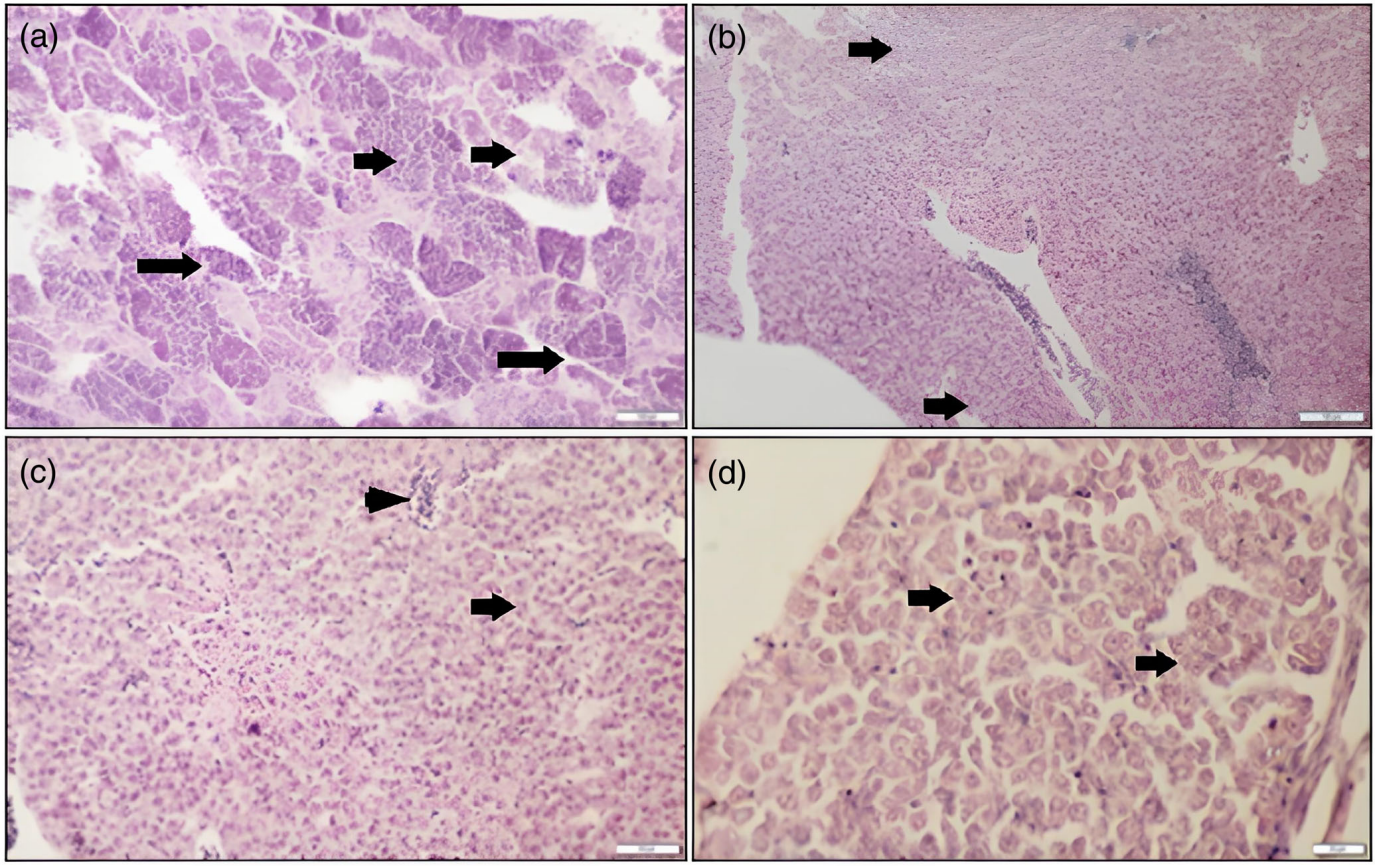
Gonad histology of *Pseudorasbora parva* male individuals. Reproductive gonads were throughout the entire season, along with spent gonads. (A) The beginning of the breeding season marks the late stage III and early stage IV. (B–D) The end of the reproductive cycle at various months. Short arrow, spermatogonium; long arrow, spermatocyte; arrowhead, spermatid.

In late April and early May, the male gonads from the Beyşehir Lake population were found to be in the late period of stage III and the early stage of stage IV (Figure 5). Stages II, V, and VI were observed in the testicles from early June to late July (Figure 5). Male individuals in Eğirdir Lake were found to be in late stage III and early stage IV, suggesting the start of the breeding period around the second half of May (Figure 5). Stages V, VI, and II were observed from the first half of October. These stages mark the end of the reproductive period (Figure 5). The testes of male individuals in the Onaç Reservoir population showed signs of late stage III and early stage IV over the period from mid‐April to mid‐May (Figure 5). The end of the breeding period for male individuals in this lake was recorded between late July and mid‐August (Figure 5).

### 3.4 Fecundity and length at first maturity, *L*_50_ (cm)

The fecundity of mature *P. parva* females from all three lakes was calculated using the gravimetric method (Table 1).

**TABLE 1:** Mature females according to age in Beyşehir Lake, Eğirdir Lake, and Onaç Reservoir *Pseudorasbora parva* populations.

Age	*n*	F ± SH	TL ± SH	W ± SH	GW ± SH
Beyşehir Lake					
I	37	1395.5 ± 144.4	6.27 ± 0.14	2.80 ± 0.18	0.25 ± 0.03
II	4	2948.6 ± 551.7	7.84 ± 0.03	5.69 ± 0.31	0.67 ± 0.16
III	7	1918.2 ± 659.3	8.45 ± 0.15	6.87 ± 0.42	0.56 ± 0.18
∑	48	2087.4 ± 451.8	7.52 ± 0.10	5.12 ± 0.30	0.49 ± 0.12
Eğirdir Lake					
I	10	1457.2 ± 365.3	6.01 ± 0.11	2.66 ± 0.10	0.38 ± 0.04
II	26	2188.2 ± 514.9	7.40 ± 0.09	4.94 ± 0.28	0.72 ± 0.11
III	9	1931.4 ± 243.2	8.98 ± 0.07	9.27 ± 0.32	1.33 ± 0.14
IV	1	1153.1 ± 000.0	9.84 ± 0.00	10.37 ± 0.00	1.33 ± 0.00
∑	46	1682.5 ± 280.9	8.06 ± 0.07	6.81 ± 0.18	0.94 ± 0.07
Onaç Reservoir					
0	12	4.25 ± 1.5	0.95 ± 0.1	0.15 ± 0.02	465.3 ± 57.90
I	4	6.09 ± 0.9	2.52 ± 0.3	0.15 ± 0.04	517.2 ± 85.70
II	1	6.71 ± 0.0	3.28 ± 0.0	0.17 ± 0.00	658.7 ± 0.00
∑	17	5.68 ± 0.8	2.25 ± 0.1	0.16 ± 0.02	547.1 ± 47.8

Abbreviations: F, fecundity (number/individual/year); GW, gonad weight (g); *n*, number of samples; SH, standard error; TL, mean total length (cm), W, weight (g).

Samples were collected from three lakes to study the size at first maturity and sex determination. For the calculations, we used 1186 fish samples. These came from three different populations: Beyşehir Lake (410 individuals with TLs between 4.06 and 11.19 cm), Eğirdir Lake (335 individuals with TLs between 4.66 and 10.72 cm), and Onaç Reservoir (212 individuals with TLs between 2.83 and 6.97 cm).

**FIGURE 6:**
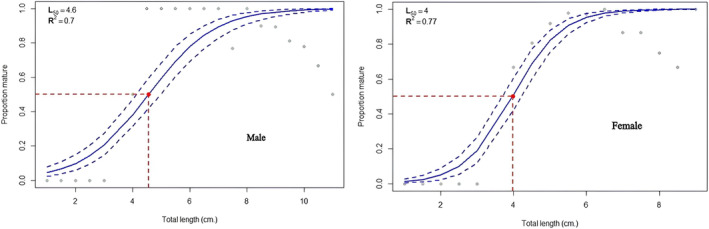
Length at first maturity of males and females in Beyşehir Lake.

**FIGURE 7:**
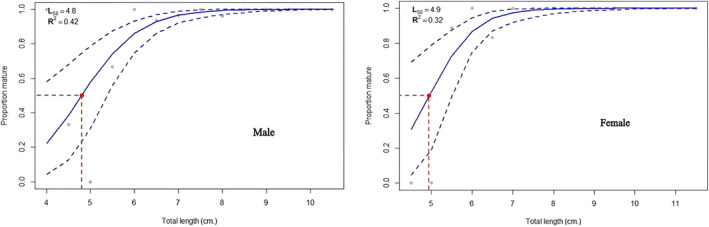
Length at first maturity of males and females in Eğirdir Lake.

**FIGURE 8:**
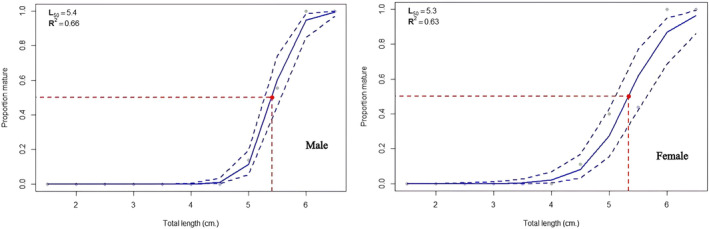
Length at first maturity of males and females in Onaç Reservoir.

Table 2 and Figures 6, 7, 8 show the variables associated with *L*_50_. The *L*_50_ lengths estimated using both Bayesian and Frequentist approaches were remarkably close to each other. Females had higher *L*_50_ values in the Eğirdir Lake population. However, males possessed greater *L*_50_ values in the Beyşehir Lake and Onaç Reservoir populations (Figures 6, 7, 8).

**TABLE 2:** Length at first maturity variables of the *Pseudorasbora parva* populations in Beyşehir Lake, Eğirdir Lake, and Onaç Reservior.

Parameters	Female			Male		
	Frequentist reg.		Bayesian reg.	Frequentist reg.		Bayesian reg.
	Original	Bootstrap	Bootstrap	Original	Bootstrap	Bootstrap
Beyşehir Lake						
*a*	−6.01	−6.1	−5.92	−3.98	−4	−3.91
*b*	1.51	1.53	1.5	0.86	0.87	0.87
*L*_50_	3.99	3.99	3.98	4.59	4.59	4.56
*R* ^2^	0.77	‐	0.77	0.7	‐	0.7
Eğirdir Lake						
*a*	−8.87	−9.05	−8.76	−7.28	−7.55	−7.27
*b*	1.77	1.79	1.79	1.5	1.55	1.51
*L*_50_	5.01	5.03	4.95	4.86	4.9	4.8
*R* ^2^	0.32	‐	0.32	0.42	–	0.42
Onaç Reservoir						
*a*	−15.22	−15.66	−15.04	−26.79	−27.3	−26.57
*b*	2.84	2.94	2.81	4.94	5.04	4.89
*L*_*50*_	5.35	5.35	5.34	5.42	5.42	5.41
*R* ^*2*^	0.62	–	0.63	0.66	–	0.66

## 4 DISCUSSION

Recent decades have witnessed the widespread translocations and introduction of alien species to new areas due to the expansion of global trade, suggesting that these species have become increasingly prevalent. From an evolutionary perspective, the widespread presence of alien species makes invasion issues deadlock. Thus, scientific research views biological invasions as significant opportunities for real‐time evolutionary experiments (Baltazar‐Soares et al., 2020). A recent study from Baltazar‐Soares et al. (2020) has examined the population structure and adaptation potential of *P. parva* in Europe and East Asia. Their study found that the populations in Slovakia and Türkiye originated from distinct regions, while a third invasion range emerged in France due to dispersion within the invasion range (Baltazar‐Soares et al., 2020).

Samples from the Sarıçay Stream (Muğla, Türkiye) showed a striking genetic similarity to the population in the Yangtze River basin in China (Baltazar‐Soares et al., 2020). In comparison with the Slovakian *P. parva* population, however, the Turkish population exhibited more allelic deviation than that between Slovakia and the Yellow River (China). The results of Baltazar‐Soares et al. (2020) reinforced the idea that the genetic structure of the population in Türkiye is not closely related to that of the Slovakian or Yellow River.

Analysis of the data from Beyşehir and Eğirdir Lakes and Onaç Reservoir showed that *P. parva* populations exhibit a high level of fecundity. Specifically, individuals in the 0+ age group were found to lay an average of 465.3 eggs, suggesting the capacity to spawn in the same year they hatch. Their mean egg production efficiency was determined as 2087 (1396–2949) in Beyşehir Lake, 1683 (1153–2188) in Eğirdir Lake, and 547 (465–659) in the Onaç Reservoir. Females in the two‐age group displayed the highest egg production across all three populations.

From assessment of the absolute and relative fecundity results (1372–1691) from the Wardynka River in Poland (Kirczuk et al., 2021), it becomes evident that the populations possess a remarkable capacity for reproduction. However, the differential outcomes may be attributed to habitat changes (rivers versus lakes) or geographical location. The total length of the examined individuals varied between 1.29 and 11.89 cm, which is significantly greater than the values documented in Turkish inland waters and Europe. In addition, the initial reproduction length, which is an important parameter for assessing reproductive potential, was measured in different populations. This length was found to be 4.0 cm in females and 4.6 cm in males in the Beyşehir Lake population, 4.9 cm in females and 4.8 cm in males in the Eğirdir Lake population, and 5.3 cm in females and 5.4 cm in males in the Onaç Reservoir population. As these findings indicate a significant reproductive potential, understanding the reproductive biology of the studied populations is vital for the management and conservation of their populations.

The age‐range values of *P. parva* samples collected from three diverse environments point to distinct findings from the population in the Wardynka River in Poland. Our study found that the age range of three different populations was narrower. However, the age range of the population in the Wardynka River was slightly larger (Kirczuk et al., 2021). The age range of our fish samples spanned from 0+ to 5 years old, which suggests that the aquatic system provides sufficient levels of nutrients for them. Anticipated demographic changes indicate a decline in the number of elderly individuals within the population, which is likely to result in increased younger individuals and reduced competition for food resources. Moreover, it signifies that the population grows as a result of the species' successful reproductive capacity and the addition of new individuals.

The observed sex ratio of females to males in our study diverged from the expected 1.00:1.00 ratio prevalent in nature (Nikolsky, 1980). Sex ratios can significantly differ among species; but many have ratios close to 1. Furthermore, it has been proposed that the dominance of one sex over the other could be attributed to differences in behavior, resulting in the easier capture of one sex and variations in mortality rates between sexes (Ghafouri et al., 2019). It is possible that the deviation from the expected male‐to‐female ratio in our study is a result of herd formation in the populations or the sampling method. The male‐to‐female ratio reported in our study is consistent with some *P. parva* populations while contradicting others (Arslan & Özeren, 2019; Benzer & Benzer, 2020a; Benzer & Benzer, 2020b). These variations may be caused by factors such as the type of hunting equipment used and the sample size.

Our research indicates that this species reaches sexual maturity at a young age (0+) and has an extended breeding period spanning 4–5 months, suggesting a significant capacity for reproduction. Additionally, the early onset of breeding in *P. parva* compared to other indigenous cyprinid species coexisting in the same environments and its ongoing ability to breed until August and even October might be regarded as evidence of its amazing adaptation.

Given the presence of abundant endemic species in the region, the invasion of *P. parva* in lakes is expected to have an adverse impact on the sensitive species. Based on what we found, it is clear that this invasive species could cause serious menace to the populations of *Pseudophoxinus hittitorum* (EN) Freyhof & Özuluğ, 2010 and *Anatolicthtyes iconii* (NT) (Akşiray, 1948) in Beyşehir Lake, *Egirdira nigra* (EN) (Kosswig & Geldiay, 1952) and *A. iconii* in Eğirdir Lake, and *Pseudophoxinus ninae* (CR) Freyhof & Özuluğ, 2006 in Onaç Reservoir. Given these observations, the affected species native to the region are classified as NT (Near Threatened), EN (Endangered), and CR (Critically Endangered) on the IUCN Red List (IUCN, 2024). Despite our observations, there was no noticeable rise in population density at Beyşehir Lake. This could be attributed to the hunting activities of pike perch in the lake. On the other hand, Eğirdir Lake, with a lower population of pike perch, might not encounter the same scenario. It is understandable from the available evidence that the region's endemic species are being impacted, falling into the NT, EN, and CR categories.

Our extensive population and observational studies conducted in the region over the past two decades provide important clues that these native species are most likely to become extinct within the next 10 years as a result of the combined impacts of drought and invasive species (Güçlü & Güçlü, 2022; Küçük et al., 2021).

This situation further highlights the remarkable growth and reproductive capacity of *P. parva* species, which has enabled it to establish a presence in numerous regions across the globe. The inevitable and expanding spread of these invasive species will have significant negative impacts on local species, particularly those that are endemic to the area. This alarming spread will lead to a further decline in global biodiversity. The presence of *P. parva* species on the remote island of Fiji, which has no connection to any major continent in the middle of Oceania, is an intriguing yet concerning situation. This emphasizes the significance of its unique, high ecological tolerance, therefore we must prioritize taking action to prevent the worldwide spread of these invasive species.

## AUTHOR CONTRIBUTIONS

FK, Conceptualization; Investigation; Writing — originaldraft; Methodology; Validation; Visualization; Writing — review & editing;Formal analysis; Project administration; Data curation; Supervision. EB, Conceptualization;Investigation; Methodology; Visualization; Validation; Data curation; Resources.SSG, Conceptualization; Investigation; Writing — original draft; Methodology;Validation; Visualization; Writing — review & editing; Formal analysis;Project administration; Data curation; Supervision. MN, Methodology;Visualization; Validation; Data curation. İG, Conceptualization;Investigation;Methodology;Visualization; Validation; Data curation. SÖKY, Investigation;Methodology; Data curation; Resources.

## CONFLICT OF INTEREST STATEMENT

The authors declare that they have no conflicts of interest.

## Data Availability

On reasonable request, the corresponding author will receive access to the article's data.
